# Prevalence, knowledge, attitude and practices of female genital mutilation and cutting (FGM/C) among United Arab Emirates population

**DOI:** 10.1186/s12905-020-00949-z

**Published:** 2020-04-22

**Authors:** Shamsa Al Awar, Moamar Al-Jefout, Nawal Osman, Zuhur Balayah, Nourah Al Kindi, Teodora Ucenic

**Affiliations:** 1 Department of Obstetrics and Gynecology, College of Medicine and Health Sciences, United Arab Emirates University, P.O. Box 15551, Al Ain, Abu Dhabi, United Arab Emirates; 2 Department of Obstetrics and Gynecology, Tawam Hospital, Al-Ain, United Arab Emirates; 3 Al Ain Hospital, Al Ain, United Arab Emirates; 4 Department of Obstetrics and Gynecology, Faculty of Medicine, Mutah University, Mu’tah, Jordan; 5 Department of Obstetrics and Gynecology №1, Sechenov First Moscow State Medical University (Sechenov University), Moscow, Russia; 6 NMC Specialty Hospital, Al Ain, United Arab Emirates

**Keywords:** Female genital mutilation/cutting, Female circumcision, UAE, Social impact, Attitude, Prevalence

## Abstract

**Background:**

Female genital mutilation/cutting (FGM/C) is a common practice in developing countries, including the UAE, and presents a major health problem.

**Methods:**

A questionnaire-based cross-sectional study was conducted among 1035 participants: 831 (80.3%) females and 204 (19.7%) males.

**Results:**

The number of women with FGM/C was 344; hence the prevalence of FGM/C in our study was 41.4%. Type I was the most prevalent (62.8%), followed by Type II (16.6%) and Type III (5%). FGM/C was less prevalent among educated and employed women (*p*-value < 0.001) and was mostly performed during infancy and childhood. Among the participants, 13.7% reported that their daughters had undergone FGM/C, with Type I being the most common, and 25% of them planned to have their future daughters undergo Type I FGM/C. While FGM/C was mostly performed by ritual circumcisers (74.4%), in 25 and 36.7% of the cases, it was performed by health professionals and in the clinic setting, respectively. About 69% of the participants considered FGM/C a custom, 72.8% were against the practice, and only 17.4% believed in its legality. Complications occurred in 30% of cases. The type of FGM/C was associated with the occurrence of complications: bleeding, difficulties in sexual life, and delivery-related problems (*p*-value < 0.05). One-fifth of the male participants expressed plans to circumcise future daughters (*p*-value < 0.001).

**Conclusion:**

FGM/C remains a prevalent practice in the UAE and has a negative association with the general health of Emirati women. The lack of clear legislation to criminalize this practice is a problem to be addressed. In this context, national-level educational and legal strategies should be a priority.

## Background

Female genital mutilation/cutting (FGM/C), defined by the World Health Organization (WHO) as “all procedures involving partial or total removal of the female external genitalia or other injury to the female genital organs for non-medical reasons,” is internationally recognized as a violation of the fundamental rights of women and girls [1]. FGM/C can be classified as: Type I—Excision of the prepuce with or without partial or total excision of the clitoris; Type II—Excision of the prepuce and clitoris together with partial or total excision of the labia minora; Type III—Excision of part or all of the external genitalia and stitching/narrowing of the vaginal opening (infibulation); Type IV—Unclassified: Pricking, piercing, or incision of the clitoris and/or labia [2]. FGM/C has no health benefits and can immediately cause severe bleeding and urination problems. Subsequent issues can include cysts, infections, and infertility, as well as complications in childbirth and increased risk of newborn deaths. FGM/C is mostly carried out on young girls between infancy and age 15. However, with the WHO and United Nations Children’s Fund (UNICEF)‘s firm stand against FGM/C based on several medical reports of its negative impact on reproductive and public health, the practice has been on the decline. Both the WHO and UNICEF strongly advocate for new legislation that can result in the banning and eventual eradication of FGM/C [3, 4].

FGM/C is a controversial topic in the Arab world, and there is no consensus on whether it is an Islamic requirement or a tribal tradition. However, the influential Egyptian Muslim institution Dar Al-Ifta Al-Misriyyah recently confirmed in a press statement that FGM/C is religiously forbidden owing to its negative impact on physical and mental well-being. Sheikh of Al-Azhar reconsiders unreliable fatwas released by some members of the faculty of Al-Azhar University (Egypt) who claim FGM/C is a religious necessity based on weak Hadith (Prophet Mohammad’s teachings) through the following statement: “This act has no religious origin, it only dates back to inherited traditions and customs and the biggest evidence for not being a religious duty for women is that the Prophet Muhammad had not circumcised his daughters.” Nevertheless, this practice is widely prevalent in many Muslim countries, especially Sudan [5], Iran [6], and Egypt [7].

FGM/C affects more than 125 million women and girls, predominantly across central Africa, parts of the Middle East and South Asia, and diaspora communities [8]. In a recent Sudanese survey of 21,947 women, of whom 6249 (28.5%) were from urban areas and 15,698 (71.5%) from rural areas, the prevalence of female circumcision was 89%. Thirty-two percent of the women had circumcised their daughters. Reported prevalence rates vary dramatically across and within countries. The highest reported prevalence rates are in Somalia (98%), Guinea (97%), Djibouti (93%), Sierra Leone (90%), and Mali (89%) [9]. FGM/C is even practiced in Western countries such as the United States owing to the rapid growth in the number of immigrants from FGM/C-practicing countries [10]. The situation is the same in the UK [11]. In the context of the UAE, a WHO study on the topic did not provide any data regarding the prevalence of FGM/C [12]. Hence, there is no reliable data about FGM/C in the UAE.

FGM/C has no medical benefits and is associated with severe health complications. In fact, it poses a serious health problem in some parts of the Arab world and is considered a harmful procedure that causes many complications during pregnancy and childbirth. During labor, FGM/C can impede delivery and cause infection and inflammation, as well as making intrapartum vaginal examination or catheterization difficult or even impossible [2]. Moreover, FGM/C may cause trauma and leave a long-lasting negative psychological impact on injured young females [13]. The consequences of this practice are not limited to women; men have described complications such as difficulty in penetration, wounds/infections on the penis, and psychological problems [14].

In 2008, the WHO, together with nine other United Nations partners, issued a new statement to advocate for the abandonment of FGM/C. The 2008 statement provides evidence about the practice collected over the past decade. It highlights the human rights and legal dimensions of the problem and provides data on the frequency and scope of FGM/C. It also summarizes research on why FGM/C continues, how to stop it, and its detrimental effects on the health of women, girls, and newborns. The new statement builds upon the 1997 original issued by the WHO, UNICEF, and United Nations Population Fund [15].

As UAE-specific data regarding FGM/C prevalence and attitude are scarce; the only single report is in a newspaper, Hence, the aim of this study was to explore the prevalence of FGM/C, attitudes toward FGM/C, and medical and social consequences of FGM/C in the UAE.

## Methods

### Setting

This study was conducted in Al Ain, Abu Dhabi city, from October 2016 to June 2017. Al Ain is located in the eastern region of Abu Dhabi Emirate and is considered the fourth largest city in the UAE with a population of 766,936 (ref. Abu Dhabi Digital Government). At 30%, Al Ain has a high Emirati national population. The recruitment sites included Al Ain Hospital (AAH) (10 outpatient clinics plus pharmacy): paper copy of questionnaire; three Fatima College of Health Sciences (FCHS) campuses (student and staff): online questionnaire; and United Arab Emirates University (UAEU) (alumni): online questionnaire. This study was approved by the Al Ain Medical District Human Research Ethics Committee in February 2016. All participants provided written or electronic informed consent for participation before the interview.

### Design

The sample size for this cross-sectional study was 1035. Separate structured/semi-structured questionnaires for female and male participants, available in both English and Arabic, were used, ensuring inclusion of both Arabic and non-Arabic speakers. The questionnaire was developed by the principal investigator by combining various similar questionnaires and validated through a small pilot study with students at the UAEU’s College of Medicine and Health Sciences. The process of development factored in social acceptance, ethnic and gender sensitivity, and applicability (supplements). The questionnaire was pre-tested on clerkship students to improve the relevance and appropriateness of the questions to the social context and subsequently revised. The interviewers underwent a two-hour training session before commencing the study. Participants answered the questionnaire in an approximately 10-min interview.

The participants were recruited from three sites—UAEU, FCHS, and AAH—and included patients, students, and university staff. Particular sites were chosen because of the high numbers of Emirati nationals and Arabs working or studying there, as well as owing to the logistics of obtaining ethical approval to recruit the appropriate sample size.

### Sampling and participant criteria

Of the 1150 individuals initially approached, the 1035 who provided complete responses were included in the study. Hence, the non-response rate was 10%. Sample size calculation was based on Kish’s formula (n0 = Z2 p q/e2) (1.96*0.3*0.9/0.0009 = 588, Z = 1.96, prevalence* (*P* = 30%), e = margin of error = 3%; *prevalence of FGM/C in Africa and Asia = 30%. Considering a non-response rate of 10%, the desired sample size was (588/0.9) or 653 respondents.

The data were collected either through face-to-face or online completion of the questionnaire at a single time point. Face-to-face data collection was undertaken at AAH (where patients attending 10 outpatient clinics including the antenatal clinic were approached) by a research assistant or interns/residents and nurses, who also offered assistance to any illiterate participants. The online questionnaire was distributed via an email link to UAEU graduates and FCHS students and staff. Consent was implied by voluntary completion of the questionnaire, following provision of study information to participants. This approach was chosen over obtaining written informed consent as it allowed completely anonymous participation and facilitated a better response rate.

Collected variables in the structured/semi-structured questionnaire included sociodemographic characteristics such as age, education, religion, marital status, ethnicity, and income, as well as FGM/C-related knowledge, attitude, practices, and complications.

The sample, which included both males and females, consisted of UAE citizens as well as non-UAE citizens. Adults aged 18 years and above, who resided in the UAE, regardless of religion, were included. The data were collected over eight months. Although the originally intended sample size was at least 2000, owing to difficulties in recruitment because of social restraints and cultural barriers, we ultimately analyzed the data of 1035 participants.

The primary objectives of the study were to determine the prevalence of FGM/C in the UAE as well as the attitudes toward the practice. The secondary objectives were to evaluate the medical and social aspects of FGM/C among women living in the UAE.

### Statistical analysis

Statistical analysis was performed using SPSS version 20 (SPSS Inc., Chicago, IL, USA). Simple frequency tables were used to describe the sample’s sociodemographic characteristics and related attitudes. Frequencies and proportions of categorical variables were compared using Pearson’s χ2 test, where appropriate. A Z-score calculator was used to measure whether two populations (circumcised women and their daughters) differed significantly on some single or categorical characteristic. The level of statistical significance was set at p-0.05.

## Results

This study was conducted between 2016 and 2017. The 1035 participants included 831 (80.3%) females and 204 (19.7%) males. The sample’s sociodemographic characteristics are depicted in Table 1.

**Table 1:** Demographic data of participants (the numerals indicate the number of participants who gave that response)

Variable	Subcategory	Number(***N***)	Percentage (%)
**Gender**	Male	204	19.7
	Female	831	80.3
**Age**	18–30 yrs.	583	56.3
	31–40 yrs.	287	27.7
	41–50 yrs.	130	12.6
	> 50 yrs.	29	2.8
**Ethnicity**	African country	55	5.3
	Arab country	268	25.9
	Asian country	42	4.1
	European country	12	1.2
	North/South America, Australia, NZ	8	0.8
	UAE	647	62.5
**Origin**	UAE	647	62.5
	GCC	24	2.3
	Other Arab Countries	191	18.5
	African country	110	10.6
	Asian country	40	3.9
	European/American/Australia/NZ	20	1.9
**Marital status**	Married	531	51.3
	Divorced	47	4.5
	Single	435	42.0
	Widow	19	1.8
	Total	1032	99.7
**Do you have children?**	No	494	47.7
	Yes	533	51.5
**Educational level**	Illiterate	10	1.0
	Primary school	42	4.1
	Secondary school	215	20.8
	University	761	73.5
**Religion**	Christian	45	4.3
	Muslim	985	95.2
	Other	3	0.3
**Employment**	Employed	419	40.5
	House wife	151	14.6
	Not-employed	68	6.6
	Student	386	37.3
**Monthly income**	Student	315	30.4
	Less < 5000 Dh	148	14.3
	5000–25,000 Dh	328	31.7
	More > 25,000 Dh	143	13.8
**Most recent Daughter Age of circumcision**	During infancy (0–1 years)	90	78.9
	Childhood (2–10 years)	20	17.6
	Adolescent (12–19 years)	3	2.6
	Adult (20 years)	1	0.9

The number of women with FGM/C was 344 out of 831; hence, the prevalence among our participants was 41.4%. Regarding the type of FGM/C performed, Type I (minimal) was the most prevalent (*n* = 216; 62.8%), followed by Type II (moderate) (*n* = 57; 16.6%) and Type III (*n* = 17; 5%). Surprisingly 1.4% of the participants were unaware of the type of FGM/C they had undergone and 14.2% did not want to answer the question (Table 2). Among all female respondents, 114 (13.7%) reported that their daughters had undergone FGM/C and about 25% were in favor of circumcision for their future daughters; the majority of these women were in favor of Type I. Regarding the type of FGM/C among participants’ daughters, Type I (*n* = 93; 81.6%) was the most common, followed by Type II (*n* = 21; 18.4%). None of the daughters had undergone Type III (Table 2). Most recent daughters’ period of circumcision was during infancy (0–1 years) (*n* = 90; 78.9%) and in most cases, the FGM/C procedure was performed in a private hospital/clinic (*n* = 84; 73.7%) (Table 3).

**Table 2:** Prevalence of FGM/C and related factors among participants and their daughters (the numerals indicate the number of participants who gave that response)

Variable	Subcategory	Number(***N***)	Percentage (%)
**Women Circumcision status**	No	487	58.6
	Yes	344	41.4
	Total	831	100.0
**Type of circumcision among women respondent*n*** **= 344**	Minimal (Type I)	216	62.8
	Moderate (Type II)	57	16.6
	Major - Pharaonic (Type III)	17	5
	Don’t know	5	1.4
	N/A (didn’t want to answer)	49	14.2
**Daughters’ Circumcised,*n*** **= 831**	N/A (didn’t want to answer)	498	59.9
	No	219	26.4
	Yes	114	13.7
	Total	831	100
**Daughters’ Type of Circumcision,*n*** **= 114**	Minimal (Type I)	93	81.6
	Moderate (Type II)	21	18.4
	Major - Pharaonic (Type III)	0	0
	Total	114	100.0

**Table 3:** Attitude, knowledge and practices towards FGM/C among participants (Numbers showing the responders number to each specific question)

Question	Answer	Number(***N***)	Percentage (%)
**Most recent Daughter Age of circumcision**	During infancy (0–1 years)	90	78.9
	Childhood (2–10 years)	20	17.6
	Adolescent (12–19 years)	3	2.6
	Adulthood (> 20 years)	1	0.9
**Who/Where did the most recent circumcision of your daughter?**	Governmental hospital/clinic	12	10.8
	Private hospital/clinic	84	73.7
	Ritual/traditional circumcisers	15	13.5
**Do you plan or prefer circumcision for your future daughters?**	No	679	74.9
	Yes	227	25.1
**Plan or preferred type of circumcision for future daughters*n*** **= 48**	Minimal (Type I)	36	75.0
	Moderate (Type II)	4	8.3
	Major - Pharaonic (Type III)	6	12.5
	Don’t Know	2	4.2
**Custom/tradition-Consider female circumcision**	No	194	18.7
	Yes	712	68.8
**Religious (Fardh / Obligatory)**	No	849	82.0
	Yes	57	5.5
**Religious (Sunna / Recommended)**	No	698	67.4
	Yes	208	20.1
**Custom Religious Obligatory Religious Recommended**	Custom	653	63.1
	Religious Obligatory	29	2.8
	Religious Recommended	159	15.4
	Custom and Religious	65	6.3
**Are you For or Against the practice of female circumcision?**	Against	664	72.8
	For	248	27.2
**FGM performed in UAE Public hospitals clinics**	Public hospitals/clinics	72	7.0
	FGM performed in UAE Private hospitals clinics	0	0
	Private hospitals/clinics	82	7.9
	FGM performed in UAE Ritual Elderly person	0	0
	Ritual/Elderly person from the community	43	4.2
**Is FGM performed in UAE**	Don’t Know	195	18.8
	Governmental hospitals/clinics	80	7.7
	Private hospitals/clinics	192	18.6
	Ritual/traditional circumcisers	41	4.0
**Do you know where FGM is performed COMBINED**	Don’t Know	552	53.3
	Other	7	0.7
	Governmental & Private hospitals/clinics	29	2.8
	Governmental hospital & Ritual person	7	0.7
	Private hospitals & Ritual person	10	1.0
	Governmental & Private & Ritual	**2**	**0.2**
**Do you think the practice of female circumcision is legal in the UAE?**	Don’t know	521	50.3
	No	233	22.5
	Yes	180	17.4

Among all respondents, the total number of circumcised daughters was 114 (13.7%), most of whom (*n* = 90; 78.9%) had undergone FGM/C during infancy. The most recent daughter circumcision was performed in private hospitals/clinics (*n* = 84; 73.7%). In contrast, among mothers, FGM/C had mostly been performed by ritual/traditional circumcisers (*n* = 232; 74.4%), followed by health professionals/at private clinics (*n* = 78; 25%), with only 117 (36.7%) reporting having been circumcised in a clean/sterile environment.

Among all the participants, 69% considered FGM/C a custom, about 5% considered it a religious ritual (fardh/obligation), and about 20% considered it a sunna/recommended act. Most respondents (*n* = 664; 72.8%) were against the practice of female circumcision, and regarding their opinion on the legality of the practice in the UAE, only 180 (17.4%) reported that they considered it legal (Table 3).

Concerning the female respondents’ preferences, only 54 (7.5%) reported that they would have voluntarily undergone FGM/C. Only 40 women (12.9%) had undergone genital repair after delivery (Table 4).

**Table 4:** Knowledge, attitude, and practice of FGM/C among female respondents (*n* = 831) (the numerals indicate the number of participants who gave that response)

Question	Answer	Number	Percentage (%)
**If you had the choice would you choose to be circumcised?**	N/A (didn’t know)	399	55.2
	No, I would not have chosen it for myself	270	37.3
	Yes, I would have chosen it again for myself	54	7.5
**Did you repair it after delivery?*n*** **= 309**	N/A i.e. have never had vaginal birth	71	22.9
	No	198	64.2
	Yes	40	12.9
**Who did the initial circumcision*n*** **= 312**	Ritual person/traditional circumcisers	232	74.4
	Health professional personnel / private clinic	78	25
	Don’t know	2	0.6
**Was it done under clean/sterile environment*n*** **= 319**	I do not recall	148	46.4
	No	54	16.9
	Yes	117	36.7
**Age of first circumcision Woman**	During infancy (0–1 years)	151	47.9
	Childhood (2–10 years)	155	49.2
	Adolescent (12–19 years)	8	2.6
	Adult (20 years)	1	0.3
**Complication pain*n*** **= 310**	Do Not recall	210	67.7
	No	56	18.1
	Yes	44	14.2
**Complication infection*n*** **= 310**	Do Not recall	210	67.8
	No	98	31.6
	Yes	2	0.6
**Complication bleeding*n*** **= 310**	Do Not recall	210	67.8
	No	74	23.9
	Yes	26	8.3
**Difficult sexual life*n*** **= 310**	Do Not recall	210	67.8
	No	91	29.4
	Yes	9	2.8
**Difficulties with deliveries*n*** **= 310**	Do Not recall	210	67.8
	No	94	30.3
	Yes	6	1.9
**Difficulties with urination*n*** **= 310**	Do Not recall	210	67.8
	No	79	25.5
	Yes	21	6.7
**Emotional distress*n*** **= 310**	Do Not recall	210	67.8
	No	87	28.1
	Yes	13	4.1
**Complications All COMBINED*n*** **= 310**	Do Not recall	210	67.8
	No	6	1.9
	Yes	94	30.3
**In what country was your circumcision performed in?*n*** **= 318**	UAE	252	79.3
	North/South America, Australia, NZ	2	0.6
	Asian country	2	0.6
	Arab country	46	14.5
	UAE	252	79.3
	African country	**16**	**5**

The association between circumcision status and educational level (illiterate, primary school, secondary school, and university) was statistically significant (*p*-value < 0.001). There was an inverse association between circumcision status and literacy level: with increasing educational levels, there was a decrease in the proportion of women with FGM/C. The association between women’s circumcision status (yes, no) and employment status was also statistically significant (*p*-value < 0.001), as the highest rates of FGM/C were among housewives and unemployed women (58.3 and 56.9%, respectively). Moreover, the association between circumcision status (yes, no) and nationality was statistically significant (*p*-value < 0.001), where the highest rate of FGM/C was among women from the Gulf Cooperation Council (GCC) nations and UAE (54.5 and 51.9%, respectively), and the lowest rate was among women from other non-African Arab countries (10.1%) (Table 5).

**Table 5:** Association between FGM/C and educational level, employment status, and nationality

		**Educational level n (%)**					***p*-value**^**a**^
		**Illiterate**	**Primary School**	**Secondary School**	**University**		
**Women Circumcision status**	Yes	7 (70.0%)	29 (69.0%)	92 (48.9%)	212 (36.2%)		<0.001
	No	3 (30.0%)	13 (31.0%)	96 (51.1%)	373 (63.8%)		
		**Employment status*n*(%)**					
		**Employed**	**House wife**	**Unemployed**	**Student**		
**Women Circumcision status**	Yes	130 (46.9%)	88 (58.3%)	33 (56.9%)	89 (26.3%)		<0.001
	No	147 (53.1%)	63 (41.7%)	25 (43.1%)	250 (73.7%)		
		**Origin, nationality: grouped by region*n*(%)**					
		**UAE**	**GCC**	**Arab country**	**African country**	**Asian country**	
**Women Circumcision status**	Yes	279 (51.9%)	12 (54.5%)	15 (10.1%)	32 (40.5%)	5 (16.1%)	<0.001
	No	259 (48.1%)	10 (45.5%)	133 (89.9%)	47 (59.5%)	26 (83.9%)	

^**a**^**Chi-square**

The individual complications reported by circumcised women included pain, infection, bleeding, difficulties in sexual life, delivery, and urination, and emotional distress at 14.2, 0.6, 8.3, 2.8, 1.9, 6.7%, and 4.1, respectively. About 30% reported all these complications. There was no significant association between all complications and genital repair after delivery (*p*-value > 0.05) (Table 6). However, the relationship between FGM/C type (minimal: Type I, moderate: Type II, and major: Type III) and the occurrence of complications (bleeding, difficulties in sexual life, and delivery-related problems, respectively) was statistically significant (*p*-value < 0.05). In addition, we observed direct trends regarding the occurrence of complications such as bleeding and delivery-related problems as the rate of complications increased from minimal-type I to moderate-type II to major pharaonic-type III of FGM/C (Table 7).

**Table 6:** Relationship between all FGM/C complications and genital repair after delivery

Complications		Did you repair it after delivery?			***p***-value^**a**^
		Have never had vaginal birth ***N*** (%)	No ***N*** (%)	Yes ***N*** (%)	
**Pain**	Do Not recall	49 (70.6)	126 (66.5)	25 (61.5)	0.445
	No	9 (11.8)	39 (20.7)	8 (20.5)	
	Yes	12 (17.6)	24 (12.8)	8 (17.9)	
**Infection**	Do Not recall	48 (70.6)	125 (66.5)	24 (61.5)	0.083
	No	18 (26.5)	63 (33.5)	15 (38.5)	
	Yes	2 (2.9)	0(.0)	0(.0)	
**Bleeding**	Do Not recall	48 (70.6)	125 (66.5)	24 (61.5)	.842
	No	15 (22.1)	47 (25.0)	10 (25.6)	
	Yes	5 (7.4)	16 (8.5)	5 (12.8)	
**Difficult sexual life**	Do Not recall	48 (70.6)	125 (66.5)	24 (61.5)	.082
	No	19 (27.9)	59 (31.4)	11 (28.2)	
	Yes	1 (1.5)	4 (2.1)	4 (10.3)	
**Difficulties with deliveries**	Do Not recall	48 (70.6)	125 (66.5)	24 (61.5)	.601
	No	19 (27.9)	60 (31.9)	13 (33.3)	
	Yes	1 (1.5)	3 (1.6)	2 (5.1)	
**Difficulties with urination**	Do Not recall	48 (70.6)	125 (66.5)	24 (61.5)	.778
	No	15 (22.1)	49 (26.1)	13 (33.3)	
	Yes	5 (7.4)	14 (7.4)	2 (5.1)	
**Emotional distress**	Do Not recall	48 (70.6)	125 (66.5)	24 (61.5)	.336
	No	15 (22.1)	57 (30.3)	14 (35.9)	
	Yes	5 (7.4)	6 (3.2)	1 (2.6)	

^**a**^**Chi-square**

**Table 7:** Relationship between FGM/C type and complications

Complications	Type of circumcision				***p***-value^a^
		Minimal (Type I)	Moderate (Type II)	Major – Pharaonic (Type III)	
**Pain**	No	29 (49.2%)	15 (60.0%)	7 (70.0%)	0.377
	Yes	30 (50.8%)	10 (40.0%)	3 (30.0%)	
**Infection**	No	59 (100.%)	23 (92.0%)	10,100.0% ()	0.060
	Yes	0 (.0%)	2 8.0% ()	0 (.0%)	
**Bleeding**	No	48 (81.4%)	18 (72.0%)	4 (40.0%)	0.020
	Yes	11 (18.6%)	7 (28.0%)	6 (60.0%)	
**Difficult Sexual Life**	No	54 (91.5%)	25 (100.0%)	6 (60.0%)	0.001
	Yes	5 (8.5%)	0 (.0%)	4 (40.0%)	
**Difficulties With Deliveries**	No	58 (98.3%)	24 (96.0%)	7 (70.0%)	0.001
	Yes	1 (1.7%)	1 (4.0%)	3 (30.0%)	
**Difficulties With Urination**	No	46 (78.0%)	20 (80.0%)	8 (80.0%)	0.973
	Yes	13 (22.0%)	5 (20.0%)	2 (20.0%)	
**Emotional Distress**	No	52 (88.1%)	20 (80.0%)	9 (90.0%)	0.573
	Yes	7 (11.9%)	5 (20.0%)	1 (10.0%)	
**Complications Combined-All**	No	4 (6.8%)	1 (4.0%)	0 (.0%)	0.638
	Yes	55 (93.2%)	24 (96.0%)	10 (100.0%)	

^a^Chi-Square

The relationship between age at circumcision and delivery-related complications was statistically significant, as the highest rate (33.3%) was among women who were circumcised in adolescence (12–19 years) (*p*-value = 0.007). However, the relationship between age at circumcision and individual complications such as pain, infection, bleeding, difficulties in sexual life and urination, and emotional distress, as well as all complications combined, was not significant (*p*-value > 0.05). Despite the absence of significant associations, we observed direct trends in the context of difficulties with urination, emotional distress, and all combined complications and age at circumcision, and it is worth mentioning that the highest rate of all complications was among those who were circumcised in adolescence (Table 8).

**Table 8:** Relationship between age at circumcision and consequent complications

Complications		Age of Woman’s First Circumcision			***p***-value^**a**^
		During Infancy (0–1 Years)	Childhood (2–10 Years)	Adolescent (12–19 Years)	
**Difficulties with Deliveries**	No	17 (89.5%)	72 (97.3%)	4 (66.7%)	0.007
	Yes	2 (10.5%)	2 (2.7%)	2 (33.3%)	
**Complications Combined**	No	2 (10.5%)	3 (4.1%)	0 (0.0%)	0.436
	Yes	17 (89.5%)	71 (95.9%)	6 (100.0%)	

^**a**^**Chi-Square**

The results show that there was a statistically significant association between men’s attitudes toward marrying circumcised women and their desire to have their future daughters circumcised, as the men who reported that marrying circumcised woman was very important to them were also the most likely to display a preference for circumcising their future daughters (21.6%) (*p*-value < 0.001). However, there was no significant association between men’s attitude toward refusing to marry uncircumcised woman and their wish to have their future daughters circumcised (*p*-value = 0.163) (Table 9).

**Table 9:** Men’s attitude toward marrying circumcised women and their desire to have their future daughters circumcised

	**Men’s attitude**	**Number & percentage*N*(%)**	**Planned or preference to circumcise future daughters**		***P*-value**^**a**^
			**No*N*(%)**	**Yes*N*(%)**	
**Importance of marrying circumcised woman**	**Not Important**	**105 (59)**	**88 (62.4)**	**17 (45.9)**	**< 0.001**
	**Slightly Important**	**31 (17.4)**	**30 (21.3)**	**1 (2.7)**	
	**Moderately Important**	**19 (10.7)**	**10 (7.1)**	**9 (24.3)**	
	**Important**	**8 (4.5)**	**6 (4.3)**	**2 (5.4)**	
	**Very Important**	**15 (8.4)**	**7 (5.0)**	**8 (21.6)**	
	**Men’s attitude**	**Number & percentage*N*(%)**	**Planned or preference to circumcise future daughters**		***P*-value**
			**No*N*(%)**	**Yes*N*(%)**	
**Refusing to marry uncircumcised woman**	**Definitely not**	**97 (56.7)**	**82 (60.3)**	**15 (42.9)**	**0.163**
	**Probably not**	**30 (17.5)**	**19 (14.0)**	**11 (31.4)**	
	**Probably**	**23 (13.5)**	**18 (13.2)**	**5 (14.3)**	
	**Very probably**	**7 (4.1)**	**6 (4.4)**	**1 (2.9)**	
	**Definitely**	**14 (8.2)**	**11 (8.1)**	**3 (8.6)**	

^**a**^**Chi-square**

## Discussion

While, at 41.4%, there was a high prevalence of FGM/C among our study participants, only 13.7% of the participants’ daughters had undergone FGM/C, which may indicate a decrease in the prevalence of the practice. This decrease, which has also been reported in other recent studies [16, 17], may be associated with several factors, perhaps the most important of which is the criminalization of FGM/C [18]. This trend has been very clear in Egypt since the 2008 criminalization of FGM/C [17]. We believe that the advances in women’s education and improvements in the awareness of health issues in the UAE may have played a role in this decrease. However, the fact that the prevalence remains high may be associated with gendered cultural forces and the continued perception of FGM/C as a potential advantage with regard to marriage prospects [19].

Type I (minimal) (62.8%) was the most prevalent, followed by Type II (moderate) (16.6%) and Type III (5%). While our data are in concordance with some studies [20], they conflict with others where Type III was the most prevalent [21]. These variations may be attributed to ethnicity-related differences in attitudes across communities. We also found that Type III FGM/C was absent among the daughters of our participants. This may be indicative of the changing attitude toward the practice owing to the high rates of complications associated with Type III FGM/C.

We found that employed and educated women were less likely to have undergone FGM/C, pointing to the high importance of female education in plans to eradicate the practice of FGM/C. Educational programs and guidelines on the management of FGM/C for health professionals [22] and the general population seem to be another important factor, as the experiences of many countries have demonstrated that FGM/C decreased after the introduction of national educational programs. It seems that FGM/C is a common deep rooted harmful traditional practice in UAE which has been kept as “TABOO” and deeply buried as untouchable secret by the influential forces of the community traditions.

About 69% of the participants considered FGM/C a custom and most respondents were against the practice. We noticed that in the context of FGM/C, there is a lack of clarity regarding the stance of religious leaders and scholars across the Muslim world. Some deny its status as a religious ritual, while others forbid Type III while accepting Type I. Nevertheless, when it comes to women, given the opportunity, the majority would neither wish to experience it themselves nor expose their daughters to the practice. This, in line with previous studies, reflects how FGM/C trends are changing [21]. However, regarding men’s attitude, it was clear that a small proportion still prefer to marry a woman with FGM/C. While Middle-Eastern countries, including the UAE, have made great strides toward gender equity, Arabic societies remain male dominated. Thus, most often, it is the men who formulate and guard society’s traditions and customs.

In the majority of the cases, FGM/C was performed during infancy (0–1 years; 48%) and childhood (2–10 years; 49%). What is alarming is that, as per the self-reports of the female respondents, they were mostly circumcised by ritual/traditional circumcisers (74.4%), with only a quarter of the women having been circumcised by a health professional/at a private clinic. Even among such cases, only a third of the circumcisions had been performed in a clean/sterile environment. These facts should be enough to alarm the authorities into taking action to ensure the safety of those who undergo this procedure secretly and in unhealthy conditions. However, we found that this trend changed over a generation, as the reverse pattern was observed among daughters: they were mostly circumcised by professionals/at private clinics (Table 3). This high percentage reflects the medicalization of FGM/C in the private sector. It is possible that the absence of clear legislation against FGM/C prompts health professionals to perform this unethical practice; hence, there is an urgent need for legislation criminalizing FGM/C.

We found that the type of FGM/C was associated with complications such as bleeding and difficulties in sexual life and delivery, with Type I being associated with the most complications. Our results are in concordance with a recent systematic review and other reports concluding that FGM/C leads to increased pain and impaired sexual satisfaction and desire [20, 23].

Additionally, we found that factors associated with the occurrence of complications, especially delivery-related difficulties, included age at FGM/C, with the greatest complications (33.3%) reported among women in the adolescent age group (12–19 years) (*p*-value = 0.007). This was also reported in Egypt [24]. We may attribute this to the fact that by this age, the female reproductive organs have almost reached peak development and any surgical procedures will be harmful and hard to correct, especially when delivery is involved.

A major strength of our study is the reasonably good sample size. Moreover, this is the first valid and large-scale survey to report on the prevalence of FGM/C among a random representative sample of the UAE population, taking into account the views of both genders, providing data regarding the intention to continue the practice at the societal level (which will be helpful guiding strategies to tackle the issue). However, an apparent weakness of our study is the fact that by being limited to Al Ain city, its results cannot be generalized to all of the UAE. In addition, not all participants answered all questions; apart from demonstrating that the topic of FGM/C is still considered a taboo, this limited our ability to procure all the necessary data. Further, women’s self-reports of having undergone FGM/C and the specific type were not verified through physical examination. Moreover, face-to-face and email interviews may have their own advantages and disadvantages, which might have contributed to the results. While we attempted to conduct the most rigorous study possible, in future research, we hope to improve the methodology and overcome the abovementioned limitations.

Even though there have been many studies in areas where the prevalence of FGM/C is high, little is known about the GCC region. Given the multinational, multicultural structure of the population in the context of the country’s legislation and advanced educational and medical system (where the practice is prohibited in governmental medical facilities), the UAE is a unique mix of tradition and modernity.

### Implications for practice and/or policy

We believe that to eradicate FGM/C in the UAE, there should be a strong and coordinated approach implemented at all levels, with an emphasis on mothers, along with the male population, health care providers, religious authorities, and legislative institutions. Of the utmost importance is the development of a national strategic plan, supportive educational programs, and targeted training programs implemented at multiple levels, such as schools, universities, and especially among health care providers and scholars in religious studies and legislation, who are uniquely positioned to support the eradication of FGM/C but are unfortunately likely to lack the necessary awareness and knowledge. We hope this study will contribute to spreading awareness about the prevalence of FGM/C and the related attitudes in the UAE. In addition, it can indicate and highlight the projected medical and social impact.

## Conclusions

FGM/C is a common practice among women in developing countries. Although there is a high prevalence of FGM/C among older generations of women in the UAE, there is a decrease in its prevalence among younger generations, especially that of Type III. Most of our participants were against FGM/C and would not prefer to undergo it if they had the choice. However, a small proportion of men still prefer to marry women with FGM/C. Urgent actions are needed to eradicate this practice. The lack of clear legislation to criminalize this practice is an important issue to be addressed, and a national educational and legal strategy to eradicate this problem should be a priority.

## Supplementary information

**Additional file 1.** Female Circumcision Study Questionnaire. FEMALE Arabic version.

**Additional file 2.** Female Circumcision Study Questionnaire FEMALE English version.

**Additional file 3:.** Female Circumcision Study Questionnaire, MALE English version.

**Additional file 4.** Female Circumcision Study Questionnaire, MALE Arabic version.

**Additional file 5.** Map 1 UAE map ((Import from wikitravel.org/shared)

## Data Availability

The data are available from the corresponding author on reasonable request. All questionnaires are available in supplementary files.

## Abbreviations

AAH: Al Ain Hospital
FCHS: Fatima College of Health Sciences
FGM/C: Female genital mutilation/cutting
GCC: Gulf Cooperation Council
WHO: World Health Organization
UAEU: United Arab Emirates University
UNICEF: United Nations Children’s Fund
