# Supplementary material for: Prevalence, knowledge, attitude and practices of female genital mutilation and cutting (FGM/C) among United Arab Emirates population
Source: BMC Womens Health. 2020 Apr 22;20:79. doi: 10.1186/s12905-020-00949-z (PMC7178722; doi:10.1186/s12905-020-00949-z)
Supplement: Supplementary file 1 — Additional file 1. Female Circumcision Study Questionnaire. FEMALE Arabic version. [file 12905_2020_949_MOESM1_ESM.pdf]

:رقم الإستبيان

استبيان الإناث

الآراء والمعتقدات والممارسات تجاه ختان الإناث في دولة الإمارات العربية المتحدة

مقدمة الدراسة: ختان الإناث

قام باحثون من قسم أمراض النساء والتوليد في جامعة الإمارات العربية المتحدة بدراسة مدى انتشار ظاهرة ختان الإناث لدى سكان دولة الإمارات العربية المتحدة وآرائهم تجاه هذه الممارسة. ونظراً لكون سكان دولة الإمارات العربية المتحدة من ثقافات وجنسيات مختلفة ومتنوعة من جهة ونظاميّ الدولة التعليمي والطبي المتقدمين من جهة أخرى فإن مجتمع الإمارات يعتبر خليطاً فريداً من العادات والتقاليد الممزوجة بالحداثة.

وتهدف هذه الدراسة البحثية لتحليل مدى انتشار الوعي والقبول تجاه ختان الإناث بين سكان دولة الإمارات العربية المتحدة حيث أن هناك القليل مما يُعرف عن مدى انتشار هذه العادة وممارساتها في منطقة الشرق الأوسط.

انتم مدعوون لاستكمال استبيان قصير لا يستغرق أكثر من 5 دقائق.

إن مشاركتكم في هذا الاستبيان ستكون سريّة للغاية ولن يتم معرفة هوية المشاركين واجاباتهم (حتى من قِبل فريق البحث) حيث أنه لن يتم جمع أي معلومات شخصية عنهم.
نرحّب بمساعدتكم ببالغ التقدير.
أشكركم على مشاركتكم

رقم الإستبيان: ☐☐☐

استبيان الإناث

العادات والتقاليد نحو ختانة الاناث في دولة الإمارات العربية المتحدة

1. العمر:
   - أ. 18 - 30
   - ب. 31-40
   - ج. 41-50
   - د. 51-60
   - ه. أكثر من 60

2. الجنسية:
   - أ. الإمارات العربية المتحدة
   - ب. دولة عربية، يرجى التحديد : ____________________
   - ج. بلد أفريقي، يرجى التحديد: ____________________
   - د. بلد أوروبي يرجى التحديد: ____________________
   - ه. بلد آسيوي يرجى التحديد: ____________________
   - و. أمريكا الجنوبية، استراليا

3. الحالة الاجتماعية
   - أ. متزوجة
   - ب. عازبة

4. هل لديكِ أطفال؟
   - أ. نعم
     - عدد الأولاد: __________
     - عدد الفتيات: __________
   - ب. لا

5. مستوى التعليم:
   - أ. جامعي
   - ب. ثانوي
   - ج. إبتدائي
   - د. لا يقرأ ولا يكتب

رقم الإستبيان:

استبيان الإناث

6. الديانة:
- أ. مسلمة
- ب. مسيحية
- ج. يهودية
- د. أخرى

7. الوظيفة:
- أ. موظفة
- ب. باحثة عن عمل
- ج. طالبة

8. الدخل الاجتماعي (الشهري):
- أ. أقل من 5000 درهم
- ب. 5000-25000 درهم
- ج. أكثر من 25000 درهم
- د. طالبة

9. هل تم ختان ابنتكِ ؟
- ا. نعم، يرجى تحديد كم من بناتكم تم ختانتهن؟ ________________
- ب. لا [إذا كانت الإجابة "لا"، يرجى الانتقال إلى السؤال 13]
- ج. لا ينطبق (لا يوجد بنات) [إذا كانت الإجابة "لا ينطبق" ، يرجى الانتقال إلى السؤال 13]

10. إذا كانت الإجابة "نعم" أي أن أحد بناتكم تم ختانتها، فما هو نوع الختان الذي استخدم؟
- أ. الحد الأدنى (نوع الأول- إزالة جزئية أو كلية للبظر و / أو القلفة)
- ب. المعتدل (النوع الثاني- إزالة جزئية أو كلية للبظر والشفرين الصغيرين، مع أو بدون استئصال الشفرين الكبيرين)
- ج. كبير (النوع الثالث- تضييق فتحة المهبل مع إنشاء غطاء عن طريق القص و الخياطة أو إزالة الشفرين الصغيرين و / أو الشفرين الكبيرين، مع أو بدون استئصال البظر (الختان الفرعوني))

11. في أي عمر قمت بختان ابنتكِ؟
- أ. في مرحلة الطفولة (0 - 1 سنة)
- ب. الطفولة (5 -11 سنوات)
- ج. المراهق. (12 - 19 سنة)
- د. سن البلوغ (≤ 20 عاما)

12. أين تم الختان؟
- أ. مستشفى حكومي / عيادة
- ب. مستشفى خاص / عيادة
- ج. الممارسة الشعبية

رقم الإستبيان: | | |

استبيان الإناث

13. هل تفكرين بختان بناتكِ في المستقبل؟
- أ. لا
- ب. نعم

إذا كانت الإجابة "نعم" ، فأي من الأنواع التالية من الختان تفكرين أو تفضلين القيام بها؟

- أ. الحد الأدنى (نوع الأول)
- ب. المعتدل (النوع الثاني)
- ج. كبير - الفرعونية (النوع الثالث)

14. هل تعتبرين ختان الإناث عادة أم طقوس دينية ؟ (يمكن اختيار أكثر من خيار واحد)
- أ. تقليدي
- ب. ديني (فرض)
- ج. ديني (سنه)

15. هل أنتِ مع أم ضد ممارسة ختان الإناث؟
- أ. مع
- ب. ضد

16. هل تعرف اين يتم القيام بختان الإناث في دولة الإمارات العربية المتحدة ؟ (يمكن اختيار أكثر من خيار واحد)
- أ. المستشفيات العامة / العيادات
- ب. المستشفيات الخاصة / العيادات
- ج. شخص مسن من المجتمع
- د. أخرى، اذكريها ....
- ه. لا أعرف

17. . هل تعتقدين أن ممارسة ختان الإناث عمل قانوني في دولة الإمارات العربية المتحدة؟
- أ. نعم
- ب. لا
- ج. لا أعرف

18. هل تم ختانكِ؟
- أ. لا *(إذا كانت اجابتك "لا" فقد انهيت هذا الاستبيان ، شكراً لكِ)*
- ب. نعم *(إذا كانت اجابتك "نعم"فالرجاء اكمال الاستبيان)*

استبيان الإناث

رقم الإستبيان: [ ] [ ] [ ]

19. هل كان لديكِ الخياربأن تختني أم لا ؟ (إذا أجبت بنعم على السؤال أعلاه)
- ◯ أ. لا، أنا لم اختره لنفسي
- ◯ ب. نعم اخترته بنفسي

في حال كنتِ قد تعرضتِ شخصياً لتجربة الختان:

20. ما هو نوع الختان الذي تعرضتِ له؟
- ◯ أ. الحد الأدنى (نوع الأول)
- ◯ ب. المعتدل (النوع الثاني)
- ◯ ج. كبير - الفرعونية (النوع الثالث)

21. هل اصلحته بعد الولادة؟
- ◯ أ. نعم
- ◯ ب. لا
- ◯ ج. لا ينطبق

22. من الذي قام بالختان:
- ◯ أ. موظف الهيئة الصحية.
- ◯ ب. الختان التقليدي.

23. هل كان ذلك في بيئة نظيفة / معقمة؟
- ◯ أ. نعم
- ◯ ب. لا
- ◯ ج. لا أذكر

24. في أي عمر تمت الختانة لديك؟
- ◯ أ. في مرحلة الطفولة (0 - 1 سنة)
- ◯ ب. الطفولة (5 -11 سنوات)
- ◯ ج. المراهقة. (12 - 19 سنة)
- ◯ د. سن البلوغ (≥ 20 عاما)

25. هل تتذكرين أي مضاعفات حصلت لك من جراء الختان؟
- ◯ أ. ألم
- ◯ ب. عدوى
- ◯ ج. نزيف
- ◯ د. صعوبات في الممارسات الجنسية
- ◯ ه. صعوبات في الولادة
- ◯ و. صعوبات في التبول
- ◯ ز. الاضطراب العاطفي

26. في أي بلد تمّت عملية الختان؟

- أ. الإمارات العربية المتحدة، يرجى ذكر المدينة ___________________________
- ب. دولة عربية، يرجى التحديد: _________________________
- ج. بلد أفريقي، يرجى التحديد: ___________________________
- د. بلد أوروبي يرجى التحديد: ___________________________
- ه. بلد آسيوي يرجى التحديد: ___________________________
- و. أمريكا الجنوبية، واستراليا

شكرا لك على المشاركة في هذه الدراسة

إن رغبتي بنسخة من الوثيقة النهائية يرجى إرسال طلبك على البريد الالكتروني :
sawar@uaeu.ac.ae
