# Supplementary material for: Prevalence, knowledge, attitude and practices of female genital mutilation and cutting (FGM/C) among United Arab Emirates population
Source: BMC Womens Health. 2020 Apr 22;20:79. doi: 10.1186/s12905-020-00949-z (PMC7178722; doi:10.1186/s12905-020-00949-z)
Supplement: Supplementary file 2 — Additional file 2. Female Circumcision Study Questionnaire FEMALE English version. [file 12905_2020_949_MOESM2_ESM.pdf]

FEMALE Questionnaire Participant Study Number: [ ][ ][ ]

Attitude, believes and practices of UAE population towards female circumcision

Survey Introduction: Female Circumcision Study

Researchers from the Obstetrics and Gynaecology Department at the UAE University are investigating prevalence of Female Circumcision in UAE population and attitude towards its practice. Given the multinational, multicultural structure of UAE population on one side, and advanced medical and education system on the other, this country is a unique mix between traditions and modern practices.

This research study aims to analyze the prevalence, awareness and acceptability towards Female Circumcision among population in the UAE since little is known about its prevalence and practices in the Middle East.

You are invited to complete a short questionnaire which should not take more than 5 minute.

Participation is confidential and anonymous which means that no one (not even the research team) will know what your answers are, as no personal identifiable details will be collected.

Your input is greatly appreciated,
Thank you for your participation.

FEMALE Questionnaire Participant Study Number: ☐☐☐

1. Age

- ◯ a. 18 – 30
- ◯ b. 31 – 40
- ◯ c. 41 – 50
- ◯ d. 51 - 60
- ◯ e. More than 60

2. Origin/nationality

- ◯ a. UAE, (please specify below which emirate)________________
- ◯ b. Arab country, *please specify*: ________________________
- ◯ c. African country, *please specify*: ________________________
- ◯ d. European country *please specify*: ________________________
- ◯ e. Asian country *please specify*: ________________________
- ◯ f. North/South America, Australia, NZ

3. Marital status

- ◯ a. Married
- ◯ b. Unmarried

4. Do you have children?

- ◯ a. Yes

  Number of Boys: ___________

  Number of Girls: ___________
- ◯ b. No

5. Level of education

- ◯ a. University
- ◯ b. Secondary school
- ◯ c. Primary school
- ◯ d. Do not read or write

FEMALE Questionnaire Participant Study Number: ☐☐☐

6. **Religion**
   - ◯ a. Muslim
   - ◯ b. Christian
   - ◯ c. Judaism
   - ◯ d. Other *Please specify*: ______________________________

7. **Employment**
   - ◯ a. Employed
   - ◯ b. Not-employed

8. **Income status (monthly)**
   - ◯ a. Less than 5000 Dh
   - ◯ b. 5000 – 25000 Dh
   - ◯ c. More than 25000 Dh
   - ◯ d. Student

9. **Is (are) your daughter(s) circumcised?**
   - ◯ a. Yes, *please specify how many of your daughters are circumcised?* _______________
   - ◯ b. No *[If No, please* ***GO*** *to* ***QUESTION 13****]*
   - ◯ c. Not applicable *(don't have any daughters)* *[If N/A, please* ***GO*** *to* ***QUESTION 13****]*

10. **If answered YES to your daughter(s) being already circumcised, what type of circumcision is already done?**
   - ◯ a. Minimal (Type I- Partial or total removal of the clitoris and/or the prepuce)
   - ◯ b. Moderate (Type II- Partial or total removal of the clitoris and the labia minora, with or without excision of the labia majora (excision))
   - ◯ c. Major - Pharaonic (Type III- Narrowing of the vaginal orifice with creation of a covering seal by cutting and appositioning the labia minora and/or the labia majora, with or without excision of the clitoris (infibulation))

11. **At what age was your most recent daughter circumcised?**
   - ◯ a. During infancy (0 – 1 years)
   - ◯ b. Childhood (5 -11 years)
   - ◯ c. Adolescent (12 – 19 years)
   - ◯ d. Adulthood (≥ 20 years)

FEMALE Questionnaire Participant Study Number: ☐☐☐

12. Who/Where did the most recent circumcision of your daughter?

- ◯ a. Governmental hospital/clinic
- ◯ b. Private hospital/clinic
- ◯ c. Ritual/traditional circumcisers

13. Do you plan or prefer circumcision for your future daughters?

- ◯ a. No
- ◯ b. Yes

  If yes, which of the following type of circumcision do you plan or prefer on doing:

  - ☐ I. Minimal (Type I)
  - ☐ II. Moderate (Type II)
  - ☐ III. Major - Pharaonic (Type III)

14. Do you consider female circumcision a custom or religious practice? *(Can choose more than one option)*

- ◯ a. Custom/tradition
- ◯ b. Religious *(Fard / Obligatory)*
- ◯ c. Religious *(Sunna / Recommended)*

15. Are you for or against the practice of female circumcision?

- ◯ a. For
- ◯ b. Against

16. **Do you know where in the UAE female circumcision is performed?** *(Can choose more than one option)*

- ◯ a. Public hospitals/clinics
- ◯ b. Private hospitals/clinics
- ◯ c. Ritual/Elderly person from the community
- ◯ d. Other, please specify: ____
- ◯ e. Don't Know

17. Do you think the practice of female circumcision is legal in the UAE?

- ◯ a. No
- ◯

b. Yes

c. Don't Know

18. Were you circumcised?

a. No [*If answered NO, that is the end of the survey, thank you*]

b. Yes [*If answered* ***"YES",*** *please* **GO** *on to complete the* ***Questions below***]

19. If you had the choice would you choose to be circumcised? *(if you answered yes to the above question)*

a. No, I would not have chosen it for myself

b. Yes, I would have chosen it again for myself

IF YOU HAD CIRCUMCISION DONE TO YOU:

20. What type of circumcision do you have?

a. Minimal (Type I)

b. Moderate (Type II)

c. Major - Pharaonic (Type III)

21. Did you repair it after delivery?

a. Yes

b. No

c. Not applicable i.e. have never had vaginal birth

22. Who did the initial circumcision?

a. Health professional personnel

b. Ritual person/traditional circumcisers

23. Was it done under clean/sterile environment?

a. Yes

b. No

c. I do not recall

FEMALE Questionnaire Participant Study Number: ☐☐☐

24. At what age you were circumcised?

- ◯ a. During infancy (0 – 1 years)
- ◯ b. Childhood (5 -11 years)
- ◯ c. Adolescent (12 – 19 years)
- ◯ d. Adulthood (≥ 20 years)

25. Do you recall any complication?

- ◯ a. Pain
- ◯ b. Infection
- ◯ c. Bleeding
- ◯ d. Difficult sexual life
- ◯ e. Difficulties with deliveries
- ◯ f. Difficulties with urination
- ◯ g. Emotional distress

26. In what country was your circumcision performed in?

- ◯ a. UAE, *please indicate city/town:* ______________________
- ◯ b. Arab country, *please specify*: ______________________
- ◯ c. African country, *please specify*: ______________________
- ◯ d. European country *please specify*: ______________________
- ◯ e. Asian country *please specify*: ______________________
- ◯ f. North/South America, Australia, NZ

Thank you for participating in this study

If you want a copy of final document please email your request to sawar@uaeu.ac.ae
