# Supplementary material for: Prevalence, knowledge, attitude and practices of female genital mutilation and cutting (FGM/C) among United Arab Emirates population
Source: BMC Womens Health. 2020 Apr 22;20:79. doi: 10.1186/s12905-020-00949-z (PMC7178722; doi:10.1186/s12905-020-00949-z)
Supplement: Supplementary file 3 — Additional file 3:. Female Circumcision Study Questionnaire, MALE English version. [file 12905_2020_949_MOESM3_ESM.pdf]

MALE Questionnaire Participant Study Number: ☐☐☐

Attitude, believes and practices of UAE population towards female circumcision

Survey Introduction: Female Circumcision Study

Researchers from the Obstetrics and Gynaecology Department at the UAE University are investigating prevalence of Female Circumcision in UAE population and attitude towards its practice. Given the multinational, multicultural structure of UAE population on one side, and advanced medical and education system on the other, this country is a unique mix between traditions and modern practices.

This research study aims to analyze the prevalence, awareness and acceptability towards Female Circumcision among population in the UAE since little is known about its prevalence and practices in the Middle East.

You are invited to <u>complete a short questionnaire which should not take more than 5 minute</u>.

Participation is confidential and anonymous which means that no one (not even the research team) will know what your answers are, as no personal identifiable details will be collected.

Your input is greatly appreciated,
Thank you for your participation.

MALE Questionnaire Participant Study Number: ☐☐☐

1. Age

- ◯ a. 18 – 30
- ◯ b. 31 – 40
- ◯ c. 41 – 50
- ◯ d. 51 - 60
- ◯ e. More than 60

2. Nationality/origin

- ◯ a. UAE
- ◯ b. Arab country, *please specify*: ______________________
- ◯ c. African country, *please specify*: ______________________
- ◯ d. European country *please specify*: ______________________
- ◯ e. Asian country *please specify*: ______________________
- ◯ f. North/South America, Australia, NZ

3. Marital status

- ◯ a. Married
- ◯ b. Unmarried

4. Do you have children?

- ◯ a. Yes
  - Number of Boys: __________
  - Number of Girls: __________
- ◯ b. No

5. Level of education

- ◯ a. University
- ◯ b. Secondary school
- ◯ c. Primary school
- ◯ d. Do not read or write

MALE Questionnaire Participant Study Number: ☐☐☐

6. **Religion**
   - ◯ a. Muslim
   - ◯ b. Christian
   - ◯ c. Judaism
   - ◯ d. Other *Please specify*: ______________________________

7. **Employment**
   - ◯ a. Employed
   - ◯ b. Not-employed

8. **Income status (monthly)**
   - ◯ a. Less than 5000 Dh
   - ◯ b. 5000 – 25000 Dh
   - ◯ c. More than 25000 Dh
   - ◯ d. Student

9. **Is (are) your daughter(s) circumcised?**
   - ◯ a. Yes, *please specify how many of your daughters are circumcised?* ______________
   - ◯ b. No *[If No, please* ***GO*** *to* ***QUESTION 13****]*
   - ◯ c. Don't Know *[If Don't Know, please* ***GO*** *to* ***QUESTION 13****]*
   - ◯ d. Not applicable *(don't have any daughters)* *[If N/A, please* ***GO*** *to* ***QUESTION 13****]*

10. **If answered YES to your daughter(s) being already circumcised, what <u>type of circumcision</u> is already done?**
   - ◯ a. Minimal (Type I- Partial or total removal of the clitoris and/or the prepuce)
   - ◯ b. Moderate (Type II-Partial or total removal of the clitoris and the labia minora, with or without excision of the labia majora (excision))
   - ◯ c. Major - Pharaonic (Type III-Narrowing of the vaginal orifice with creation of a covering seal by cutting and appositioning the labia minora and/or the labia majora, with or without excision of the clitoris (infibulation))
   - ◯ d. Don't Know

MALE Questionnaire Participant Study Number: ☐☐☐

11. At what age was your most recent daughter circumcised?

- ◯ a. During infancy (0 – 1 years)
- ◯ b. Childhood (5 -11 years)
- ◯ c. Adolescent (12 – 19 years)
- ◯ d. Adulthood (≥ 20 years)

12. Who/ Where did the most recent circumcision of your daughter?

- ◯ a. Governmental hospital/clinic
- ◯ b. Private hospital/clinic
- ◯ c. Ritual/traditional circumcisers
- ◯ d. Don't Know

13. Do you plan or prefer circumcision for your future daughters?

- ◯ a. No
- ◯ b. Yes

If yes, which of the following type of circumcision do you plan or prefer on doing:

- ☐ I. Minimal (Type I)
- ☐ II. Moderate (Type II)
- ☐ III. Major - Pharaonic (Type III)

14. Do you belief the reason for the practice of female circumcision to be: *(Can choose more than one option)*

- ◯ a. Custom/tradition
- ◯ b. Religious (Fard/Obligatory)
- ◯ c. Religious (Sunna/Recommended)

15. Are you for or against the practice of female circumcision?

- ◯ a. For
- ◯ b. Against

16. How important is marrying a circumcised woman to you?

- ◯ 1. Not Important
- ◯ 2. Slightly Important
- ◯ 3. Moderately Important
- ◯ 4. Important
- ◯ 5. Very Important

17. Would you refuse to marry an uncircumcised woman?

◯ 1. Very Unlikely
◯ 2. Unlikely
◯ 3. Somewhat likely
◯ 4. Likely
◯ 5. Very likely

18. Do you know where in the UAE female circumcision is performed? *(can choose more than one option)*

◯ a. Public hospitals/clinics
◯ b. Private hospitals/clinics
◯ c. Ritual/traditional circumcisers/elderly person from the community
◯ d. Other, please specify: ______________________________

◯ e. Don't Know

19. Do you think the practice of female circumcision is legal in the UAE?

◯ a. Yes
◯ b. No

◯ c. Don't Know

Thank you for participating in this study

If you want a copy of final document please email your request to sawar@uaeu.ac.ae
