# Supplementary material for: Prevalence, knowledge, attitude and practices of female genital mutilation and cutting (FGM/C) among United Arab Emirates population
Source: BMC Womens Health. 2020 Apr 22;20:79. doi: 10.1186/s12905-020-00949-z (PMC7178722; doi:10.1186/s12905-020-00949-z)
Supplement: Supplementary file 4 — Additional file 4. Female Circumcision Study Questionnaire, MALE Arabic version. [file 12905_2020_949_MOESM4_ESM.pdf]

رقم الإستبيان:

استبيان الذكور

الآراء والمعتقدات والممارسات تجاه ختانة الإناث في دولة الإمارات العربية المتحدة

مقدمة الدراسة: ختان الإناث

قام باحثون من قسم أمراض النساء والتوليد في جامعة الإمارات العربية المتحدة بدراسة مدى انتشار ظاهرة ختان الإناث لدى سكان دولة الإمارات العربية المتحدة وآرائهم تجاه هذه الممارسة. ونظراً لكون سكان دولة الإمارات العربية المتحدة من ثقافات وجنسيات مختلفة ومتنوعة من جهة ونظاميّ الدولة التعليمي والطبي المتقدمين من جهة أخرى فإن مجتمع الإمارات يعتبر خليطاً فريداً من العادات والتقاليد الممزوجة بالحداثة.

وتهدف هذه الدراسة البحثية لتحليل مدى انتشار الوعي والقبول تجاه ختان الإناث بين سكان دولة الإمارات العربية المتحدة حيث أن هناك القليل مما يُعرف عن مدى انتشار هذه العادة وممارساتها في منطقة الشرق الأوسط.

انتم مدعوون لاستكمال استبيان قصير لا يستغرق أكثر من 5 دقائق.

إن مشاركتكم في هذا الاستبيان ستكون سريّة للغاية ولن يتم معرفة هوية المشاركين واجاباتهم (حتى من قِبل فريق البحث) حيث أنه لن يتم جمع أي معلومات شخصية عنهم.
نرحّب بمساعدتكم ببالغ التقدير.
أشكركم على مشاركتكم

رقم الإستبيان: ☐☐☐ استبيان الذكور

العادات والتقاليد نحو ختانة الاناث في دولة الإمارات العربية المتحدة

1. العمر:
   - ◯ أ. 30-18
   - ◯ ب. 40-31
   - ◯ ج. 50-41
   - ◯ د. 60-51
   - ◯ ه. أكثر من 60

2. الجنسية:
   - ◯ أ. الإمارات العربية المتحدة
   - ◯ ب. دولة عربية، يرجى التحديد : ________________________
   - ◯ ج. بلد أفريقي، يرجى التحديد: ________________________
   - ◯ د. بلد أوروبي يرجى التحديد: ________________________
   - ◯ ه. بلد آسيوي يرجى التحديد: ________________________
   - ◯ و. أمريكا الجنوبية، استراليا

3. الحالة الاجتماعية
   - ◯ أ. متزوج
   - ◯ ب. عازب

4. هل لديكِ أطفال؟
   - ◯ أ. نعم
     - عدد الأولاد: ____________
     - عدد الفتيات: ____________
   - ◯ ب. لا

5. مستوى التعليم:
   - ◯ أ. جامعي
   - ◯ ب. ثانوي
   - ◯ ج. إبتدائي
   - ◯ د. لا يقرأ ولا يكتب

6. الديانة:
- أ. مسلمة
- ب. مسيحية
- ج. يهودية
- د. أخرى

7. الوظيفة:
- أ. موظف
- ب. باحث عن عمل

8. الدخل الاجتماعي (الشهري):
- أ. أقل من 5000 درهم
- ب. 5000-25000 درهم
- ج. أكثر من 25000 درهم
- د. طالب

9. هل تم ختان بناتكِ ؟
- ا. نعم، يرجى تحديد كم من بناتكم تم ختانتهن؟ ________________
- ب. لا [إذا كانت الإجابة "لا"، يرجى الانتقال إلى السؤال 13]
- ج. لا ينطبق (لا يوجد بنات) [إذا كانت الإجابة "لا ينطبق" ، يرجى الانتقال إلى السؤال 13]

10. إذا كانت الإجابة "نعم" أي أن أحد بناتكم تم ختانتها، فما هو نوع الختان الذي استخدم؟
- أ. الحد الأدنى (نوع الأول- إزالة جزئية أو كلية للبظر و / أو القلفة)
- ب. المعتدل (النوع الثاني- إزالة جزئية أو كلية للبظر والشفرين الصغيرين، مع أو بدون استئصال الشفرين الكبيرين)
- ج. كبير - (النوع الثالث- - تضييق فتحة المهبل مع إنشاء غطاء عن طريق القص و الخياطة أو إزالة الشفرين الصغيرين و / أو الشفرين الكبيرين، مع أو بدون استئصال البظر (الختان الفرعوني))
- د. لا أعرف

11. في أي عمر قمت بختان ابنتكِ؟
- أ. في مرحلة الطفولة (0 - 1 سنة)
- ب. الطفولة (5 -11 سنوات)
- ج. المراهق. (12 - 19 سنة)
- د. سن البلوغ (≥ 20 عاما)

رقم الإستبيان: ☐☐☐

استبيان الذكور

12. أين تم الختان؟

- ◯ أ. مستشفى حكومي / عيادة
- ◯ ب. مستشفى خاص / عيادة
- ◯ ج. ممارس شعبي
- ◯ د. لا أعرف

13. هل تفكر بختان بناتكِ في المستقبل؟

- ◯ أ. لا
- ◯ ب. نعم

إذا كانت الإجابة "نعم" ، فأي من الأنواع التالية من الختان تفكرأو تفضل القيام به؟

- ☐ أ. الحد الأدنى (نوع الأول)
- ☐ ب. المعتدل (النوع الثاني)
- ☐ ج. كبير - الفرعونية (النوع الثالث)

14. هل تعتبر ختان الإناث عادة أم طقوس دينية ؟ (يمكن اختيار أكثر من خيار واحد)

- ◯ أ. عادات و تقاليد
- ◯ ب. ديني (فرض)
- ◯ ج. ديني (سنه)

15. هل أنتِ مع أم ضد ممارسة ختان الإناث؟

- ◯ أ. مع
- ◯ ب. ضد

16. ما مدى أهمية الزواج من امرأة مختتنة بالنسبة لك؟

- ◯ 1. غير مهم
- ◯ 2. هام قليلا
- ◯ 3. هام معتدل
- ◯ 4. هام
- ◯ 5. هام جدا

17. هل ترفض الزواج من امرأة غير مختتنة ؟

- ◯ 1. مستبعد جدا
- ◯ 2. غير محتمل
- ◯ 3. مرجح إلى حد ما
- ◯ 4. محتمل
- ◯ 5. مرجح جدا

استبيان الذكور

:رقم الإستبيان

18. هل تعرف اين يتم القيام بختان الإناث في دولة الإمارات العربية المتحدة ؟ (يمكن اختيار أكثر من خيار واحد)

- ◯ أ. المستشفيات العامة / العيادات
- ◯ ب. المستشفيات الخاصة / العيادات
- ◯ ج .شخص مسن من المجتمع
- ◯ د. أخرى، اذكريها ....
- ◯ ه. لا أعرف

19. . هل تعتقد أن ممارسة ختان الإناث عمل قانوني في دولة الإمارات العربية المتحدة؟

- ◯ أ. نعم
- ◯ ب. لا
- ◯ ج. لا أعرف

شكرا لك على المشاركة في هذه الدراسة

إن رغبت في نسخة من الوثيقة النهائية يرجى إرسال طلبك على البريد الالكتروني:
sawar@uaeu.ac.ae
