# Supplementary figures and images for: Prevalence, knowledge, attitude and practices of female genital mutilation and cutting (FGM/C) among United Arab Emirates population
Source: BMC Womens Health. 2020 Apr 22;20:79. doi: 10.1186/s12905-020-00949-z (PMC7178722; doi:10.1186/s12905-020-00949-z)

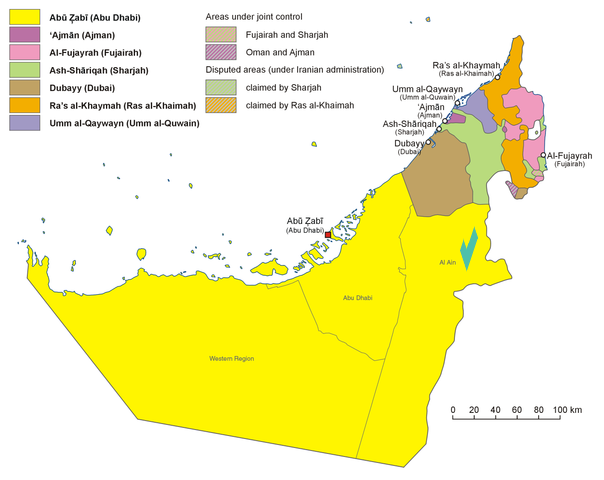

Supplement: Supplementary file 5 — Additional file 5. Map 1 UAE map ((Import from wikitravel.org/shared) [file 12905_2020_949_MOESM5_ESM.png]
